# Identification of hub genes-based predictive model in hepatocellular carcinoma by robust rank aggregation and regression analysis

**DOI:** 10.7150/jca.52089

**Published:** 2021-01-30

**Authors:** Di Wu, Yun Pan, Xueyong Zheng

**Affiliations:** 1 Department of General Surgery, Sir Run-Run Shaw Hospital, School of Medicine, Zhejiang University, Hangzhou, 310016, China.; 2 Department of Emergency, Sir Run-Run Shaw Hospital, School of Medicine, Zhejiang University, Hangzhou, 310016, China.

**Keywords:** hepatocellular carcinoma, bioinformatic analysis, regression analysis, hub gene, prognostic risk model, validation dataset

## Abstract

**Background:** Though various hub genes for HCC have been identified in decades, the limited sample size, inconsistent bioinformatic analysis methods and lacking evaluation in validation cohorts would make the results less reliable, novel biomarkers and risk model for HCC prognosis are still urgently desired.

**Methods:** The Robust Rank Aggression method was applied to integrate 12 HCC microarray datasets to screen for robustly and stably differentially expressed candidates. The Least Absolute Shrinkage and Selection Operator regression and multivariate Cox regression analysis were performed to construct a six hub genes-based prognostic model, which was further verified in matched tumor and non-tumor hepatic samples and two independent validation cohorts.

**Results:** Six hub genes for HCC were identified including CD163, EHHADH, KIAA0101, SLC16A2, SPP1 and THBS4. The risk score according to hub genes-based prognostic model could be an independent predictive factor for HCC. Quantitative real-time polymerase chain reaction results showed significant difference in expression level between tumor and non-tumor hepatic tissues. Prognostic value of risk model has been verified in TCGA-HCC and GSE76240 datasets. Biological function analysis revealed these hub genes were closely associated with tumorigenesis processes.

**Conclusion:** A novel six hub genes predictive risk model for HCC has been established based on multiple datasets analyses, providing novel features for the prediction of HCC patients' outcome.

## Introduction

Hepatocellular carcinoma (HCC), ranking the sixth in incidence among malignancies, is a widespread disease that causes over 800,000 deaths per year 1. Although alpha fetoprotein (AFP) value has been widely utilized for the diagnosis and prediction of HCC, however, the AFP levels might also be elevated in benign liver disease 2-4 and the different AFP cut-off values would possibly result in high false-positive or negative rates 5.

Recently, with the rapid development and popularization of high-throughput microarrays and sequencing technologies, more and more novel biomarkers and therapeutic targets for HCC have been reported. However, the limited sample size, inconsistent microarray platforms and different bioinformatic analyzing methods generate heterogeneity, making results greatly varied in each individual study. Therefore, predictive biomarkers or functional genes should be screened in discovery datasets with enlarged sample size by applying modified analyzing methods to generate more reliable and accurate results.

In the present study, 12 HCC microarray datasets from Gene Expression Omnibus (GEO) were analyzed using the Robust Rank Aggregation (RRA) method to identify the most stably differentially expressed genes (DEGs) between HCC samples and normal hepatic samples. These DEGs were then sequentially analyzed by the Least Absolute Shrinkage and Selection Operator (LASSO) regression method and multivariate Cox regression method to identify the hub genes with the best prognostic value in HCC. Six functional genes including CD163, EHHADH, KIAA0101, SLC16A2, SPP1 and THBS4 were filtered out. Tumor and paired non-tumor samples of 12 patients were applied to confirm the six genes' expression value. Gene Set Enrichment Analysis (GSEA) and weighted gene co-expression network analysis (WGCNA) were further conducted to investigate their potential biological functions. In addition, two HCC datasets with survival statistics, TCGA-HCC dataset and GSE76427, were used to validate the prognostic value of the hub genes-based model. The primary objective of the current research was to construct a prognostic model based on the robustly and stably expressed hub genes in HCC which could be used for predicting patients' outcome and providing potential therapeutic targets.

## Materials and methods

### Selection of HCC gene expression datasets

All microarray datasets were downloaded from GEO, the selection criteria of which were listed as follows: 1) Samples contained in datasets were diagnosed with HCC, other benign hepatic hyperplasia or non-HCC malignancies were excluded. 2) Inclusion of datasets containing HCC and normal hepatic samples. 3) Microarray platform contained more than 5000 genes. According to the above-mentioned screening criteria, 11 datasets were finally included in the current research for further analyzation, including GSE25097, GSE39791, GSE46408, GSE57957, GSE62232, GSE64041, GSE75271, GSE76427, GSE84005, GSE84402, GSE14520. For GSE14520, the dataset contained two groups measured by different platforms, GPL571 and GPL3921. Therefore, datasets analyzed by GPL571 platform and GPL3921 platform were processed independently and listed as two individual cohorts in RRA analysis.

### Identification of robustly DEGs from multiple GEO datasets

The R package “limma” was utilized for data normalization and DEGs identification in HCC. Then RRA algorithm was used to integrate the results from those 12 datasets and arrange the genes in descending order according to the adjusted P value and log_2_fold change value to screen for the most significantly upregulated and downregulated genes. DEGs with adjusted P < 0.05 and log_2_|fold change| > 1 were considered as the potential candidates for following analyses.

### Functional enrichment analyses

Gene Ontology (GO) enrichment and Kyoto Encyclopedia of Genes and Genomes (KEGG) pathway analyses were conducted using the comprehensive and simple R package “clusterprofiler” 6. GO terms and KEGG pathways with P < 0.05 or adjusted P < 0.05 were considered statistically significant and selected for visualization.

### Construction of hub genes-based predictive model in HCC

The GEO dataset GSE14520 which also provided relevant clinical information was chosen as the training dataset. After combining the two groups contained in GSE14520 into one single dataset, batch normalization using R package “limma” and “SVA” were performed to normalize the integrated data. LASSO regression using R package “glmnet” was performed on the candidates generated from RRA analysis. The results from LASSO regression analysis were further analyzed by stepwise multivariate Cox regression analysis using R package “survminer” to establish the final hub genes-based prognostic model for HCC. Risk score of each HCC patient was calculated according to multivariate Cox regression analysis.

### Validation of the hub genes and prognostic model

The expression levels of the screened hub genes were evaluated by quantitative real-time polymerase chain reaction (PCR) assay in tumor and paired non-tumor tissues from 12 HCC patients. To assess the hub genes' prognostic value, receiver operating characteristic (ROC) curves and calculated area under the ROC curve (AUC) were plotted with R package “pROC”, survival analysis was performed using R packages “survival” and “survminer”. HCC patients were divided into high- and low-risk groups using the median risk score as the cut-off value. Chi-square test was performed to compare the distribution of each clinical characteristic between two risk groups. Univariate and multivariate Cox regression analyses were performed to evaluate the prognostic value of the risk score and other clinical pathological characteristics. Similar analyses based on the six hub genes risk model were also performed in two validation cohorts including TCGA-HCC dataset and GSE76427 to determine the hub genes' prognostic value.

### Gene set enrichment analysis

The expression matrix of GSE14520 was divided into high- and low-expression groups according to each single hub gene's expression level, using median expression value as the cut-off point. Then gene set enrichment analysis was performed between the two groups by GSEA software (Version 4.0.3). P<0.05 was regarded as statistically significant. “h.all.v7.0.symbols.gmt” which was download from the Molecular Signature Database was selected as the reference gene set.

### Weighted gene co-expression network analysis

The R package “WGCNA” was applied to perform WGCNA in GSE14520 to generate clinical traits-related modules. After transforming the adjacency matrix into topological overlap matrix (TOM), genes were classified into different modules according to the TOM-based dissimilarity measure. Here, we set soft-thresholding power as 4 (scale free R^2^ = 0.85), 0.25 as cutting height and minimal module size as 30 to identify key modules. Modules containing the screened hub genes were further analyzed through GO and KEGG analyses to explore potential biological functions.

### RNA isolation and quantitative real-time PCR

RNA was isolated from tissues using RNeasy Mini Kit (Qiagen, Valencia, Canada) according to the manufacturer's protocol. One microgram of total RNA was reverse-transcribed to cDNA by Hifair^®^ III 1st Strand cDNA Synthesis Kit (Yeason, Shanghai, China). The experimental protocol was gDNA removal (42 °C for 2 minutes), followed by reverse transcription (25 °C for 5 minutes, 42 °C for 30 minutes, 85 °C for 5 minutes). Quantitative real-time PCR by a 20 µL reaction volume was performed using LightCycler^®^ 480 Real-Time PCR system. The amplification program was repeated for 40 cycles. The expression of each gene was calculated using 2^-ΔΔCT^ method. Results were normalized against the level of β-actin. Primers were designed online and purchased from Tsingke (Hangzhou, China). All primers were listed in Table 2. All the assays were performed in triplicates and results were plotted as mean ± SD.

### Clinical samples collection

Tumor and matched non-tumor samples used in the present research were obtained from 12 cases of HCC patients with completely informed consent who underwent surgical resection at Sir Run-Run Shaw Hospital. The samples derived from patients who underwent preoperative chemotherapy or radiotherapy was excluded. The study was approved by the Clinical Research Ethics Committee of Sir Run-Run Shaw Hospital of Zhejiang University.

### Statistical analysis

The Chi-square test or Student's t test (two-tailed) was used as required to analyze the statistical differences between groups using R software (Version 3.7). Figures were generated by using R software or GraphPad Prism (Version 7). Network was constructed by Cytoscape software (Version 3.7.2). P value or adjusted P value less than 0.05 was considered statistically significant. * denotes a statistical significance (* P < 0.05, ** P < 0.01, *** P < 0.001).

## Results

### Identification of robustly and stably DEGs by RRA method

**Figure 1:**
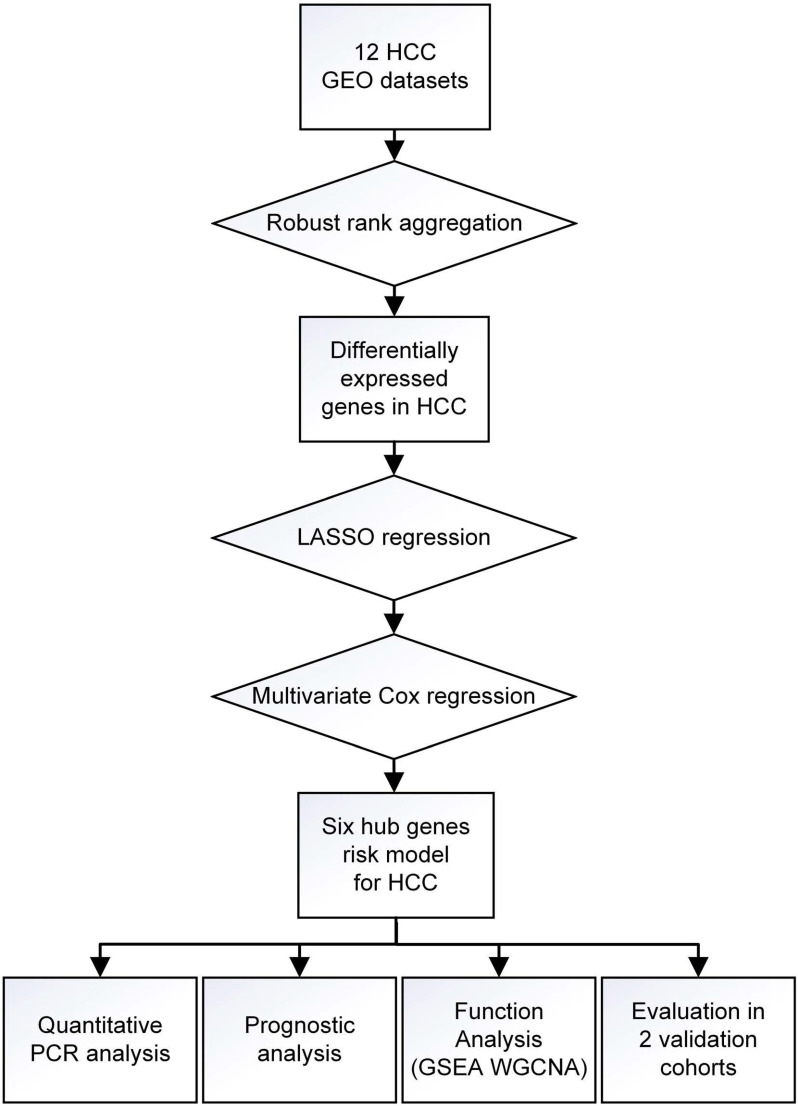
Flow chart of overall design of the present study.

**Figure 2:**
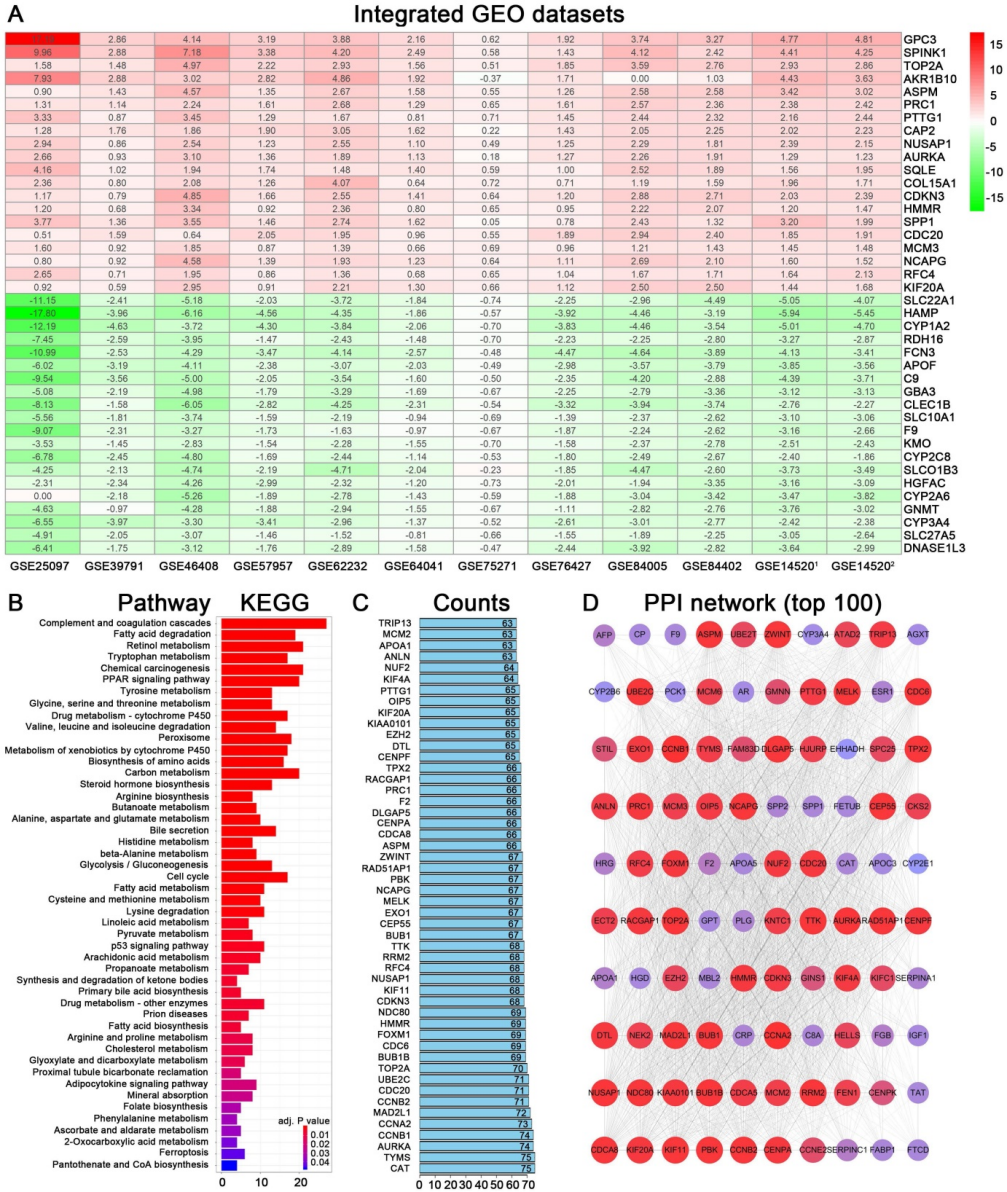
Identification of DEGs from multiple GEO datasets and functional analyses. (A) Heatmap showing the top 20 up-regulated and top 20 down-regulated DEGs according to P value and log_2_|fold change|. Each row indicated one gene and each column represents one included dataset. Red represents up-regulation and green represents down-regulation. The values in heatmap represent logarithmic fold change between tumor and normal tissues. (B) KEGG pathway enrichment of DEGs obtained from RRA method. (C) Protein-protein interaction number of DEGs ranking in top 50. (D) Protein-protein interaction network based on DEGs with interactions ranking in top 100.

A workflow of the present research for identification, validation and functional analyses of hub genes in HCC was presented in Figure 1. 11 HCC datasets met the inclusion criteria were selected for further investigation, including GSE25097, GSE39791, GSE46408, GSE57957, GSE62232, GSE64041, GSE75271, GSE76427, GSE84005, GSE84402, GSE14520. The two cohorts contained in GSE14520 which were respectively measured by platform GPL571 and GPL3921 were independently listed as GSE14520^1^ and GSE14520^2^. Therefore, a total of 12 HCC datasets were enrolled for the following DEGs screening. Brief introductions for each dataset, including GEO accession number, contactor, platform, research city, sample size, submission time were presented in Table S1. Data normalization was performed for each included cohort. Based on the results of RRA analysis, 133 up- and 426 down-regulated significant DEGs were identified. GPC3 was the most significantly up-regulated gene (adjusted P = 1.21E-32, log_2_fold change = 4.38), followed by SPINK1 (adjusted P = 1.19E-25, log_2_fold change = 3.94) and AKR1B10 (adjusted P = 3.04E-23, log_2_fold change = 2.82). Meanwhile, SLC22A1 (adjusted P = 1.44E-30, log_2_fold change = -3.83), HAMP (adjusted P = 6.08E-27, log_2_fold change = -5.18) and CYP1A2 (adjusted P = 1.54E-26, log_2_fold change = -4.41) were the top three significantly down-regulated genes. The top 40 DEGs including 20 up-regulated and 20 down-regulated genes were shown in the heatmap (Figure 2A). The heatmap for these 40 DEGs was also drawn based on TCGA-HCC dataset (Figure S1A), demonstrating a similar expression variation tendency.

### The mRNA expression of six hub genes in HCC and normal tissues

Expression value of CD163, EHHADH, KIAA0101, SLC16A2, SPP1, THBS4 were determined using quantitative real-time PCR between HCC and normal hepatic tissue samples. As shown in Figure S2, KIAA0101, SPP1 and THBS4 were significantly upregulated while EHHADH, CD163, SLC16A2 were significantly downregulated in HCC tissues compared to matched normal tissues, which were in consistent with results from microarrays or sequencing method.

### Functional enrichment analyses of DEGs in HCC

The 559 DEGs obtained from RRA analysis were chosen to perform KEGG analysis and construct protein association network. For KEGG pathway analysis, several crucial signaling pathways involved in tumorigenesis such as “chemical carcinogenesis”, “PPAR signaling pathway”, “drug metabolism-cytochrome P450” and “cell cycle” were found significantly associated with these screen DEGs (Figure 2B). The functional protein association networks for the DEGs were established by an online tool “String”. The number of interactions for each gene was calculated (Figure 2C). DEGs with interactions ranking in top 100 were selected to establish a protein-protein interaction network (Figure 2D).

### Construction of a hub genes-based prognostic model

**Figure 3:**
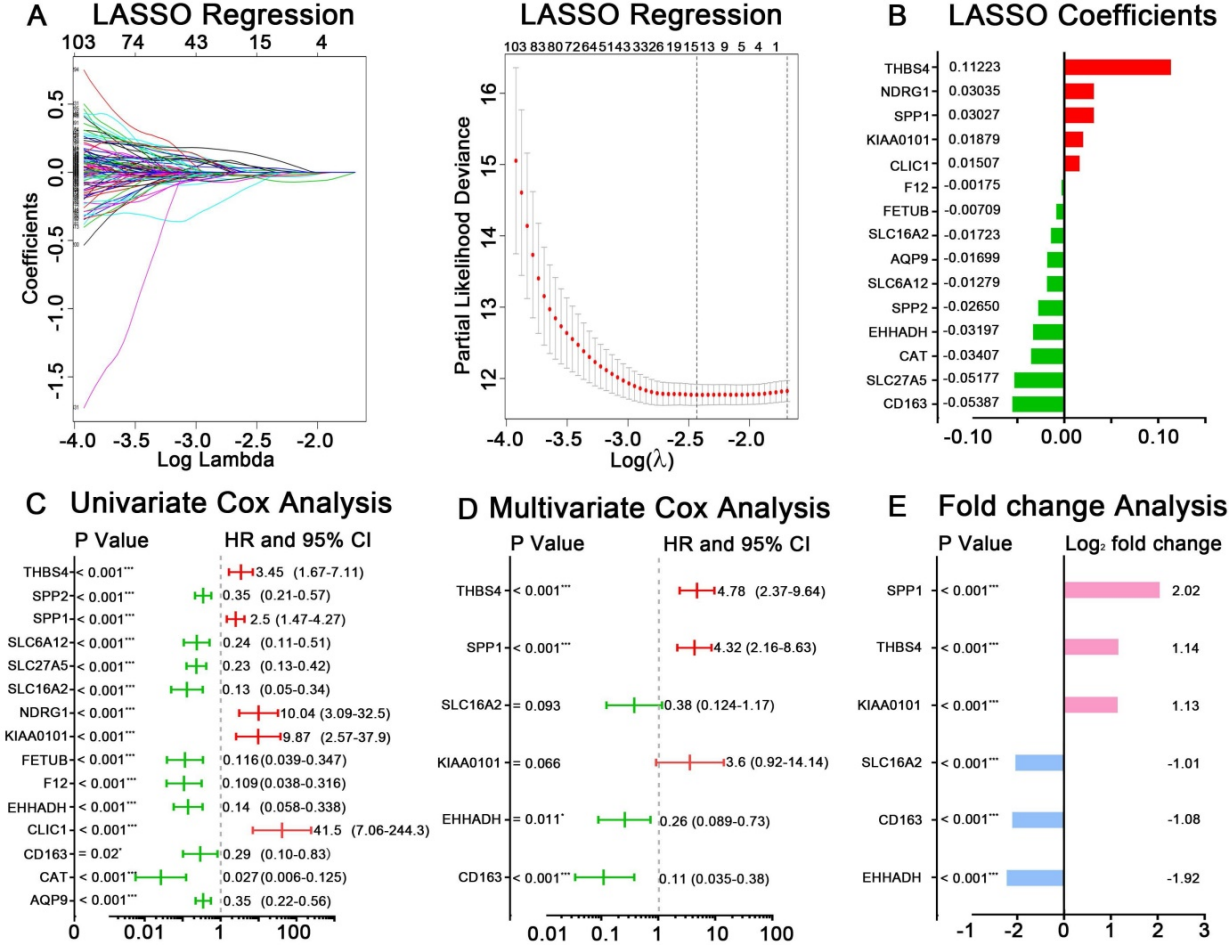
Identification of hub genes and construction of risk score model in GSE14520. (A) LASSO regression analysis of DEGs acquired by RRA methods. (B) The reserved 15 candidates from LASSO regression analysis and corresponding coefficients. (C) Univariate Cox regression analysis for 15 candidates from LASSO regression analysis. (D) Multivariate Cox regression analysis for selecting final hub genes. The hazard ratios, 95% confidence intervals and P values are shown. (E) Logarithmic fold change of the six hub genes between tumor and normal tissues based RRA result.

GSE14520 was chosen as the training dataset. By performing LASSO regression analysis, 15 candidate genes were left, including AQP9, CAT, CD163, CLIC1, EHHADH, F12, FETUB, KIAA0101, NDRG1, SLC16A2, SLC27A5, SLC6A12, SPP1, SPP2 and THBS4. The LASSO coefficient of each gene was also calculated and presented (Figure 3A-B). After applying univariate Cox regression and stepwise multivariate Cox regression analyses on the 15 seed genes, six hub genes were identified to be significantly related to HCC prognosis, which were CD163, EHHADH, KIAA0101, SLC16A2, SPP1, THBS4 (Figure 3C-D). The log_2_ fold change value of each gene according to RRA result and TCGA-HCC dataset were present in Figure 3E and Figure S1B. The predictive risk model based on the six hub genes expression value and multivariate Cox coefficients was constructed as follow: risk score = (-2.17× the expression value of CD163) + (-1.37× the expression value of EHHADH) + (1.28× the expression value of KIAA0101) + (-0.96× the expression value of SLC16A2) + (1.46× the expression value of SPP1) + (1.57× the expression value of THBS4). The detailed analyzing results for the six hub genes were listed in Table 1.

### Prognostic value of six hub genes

**Figure 4:**
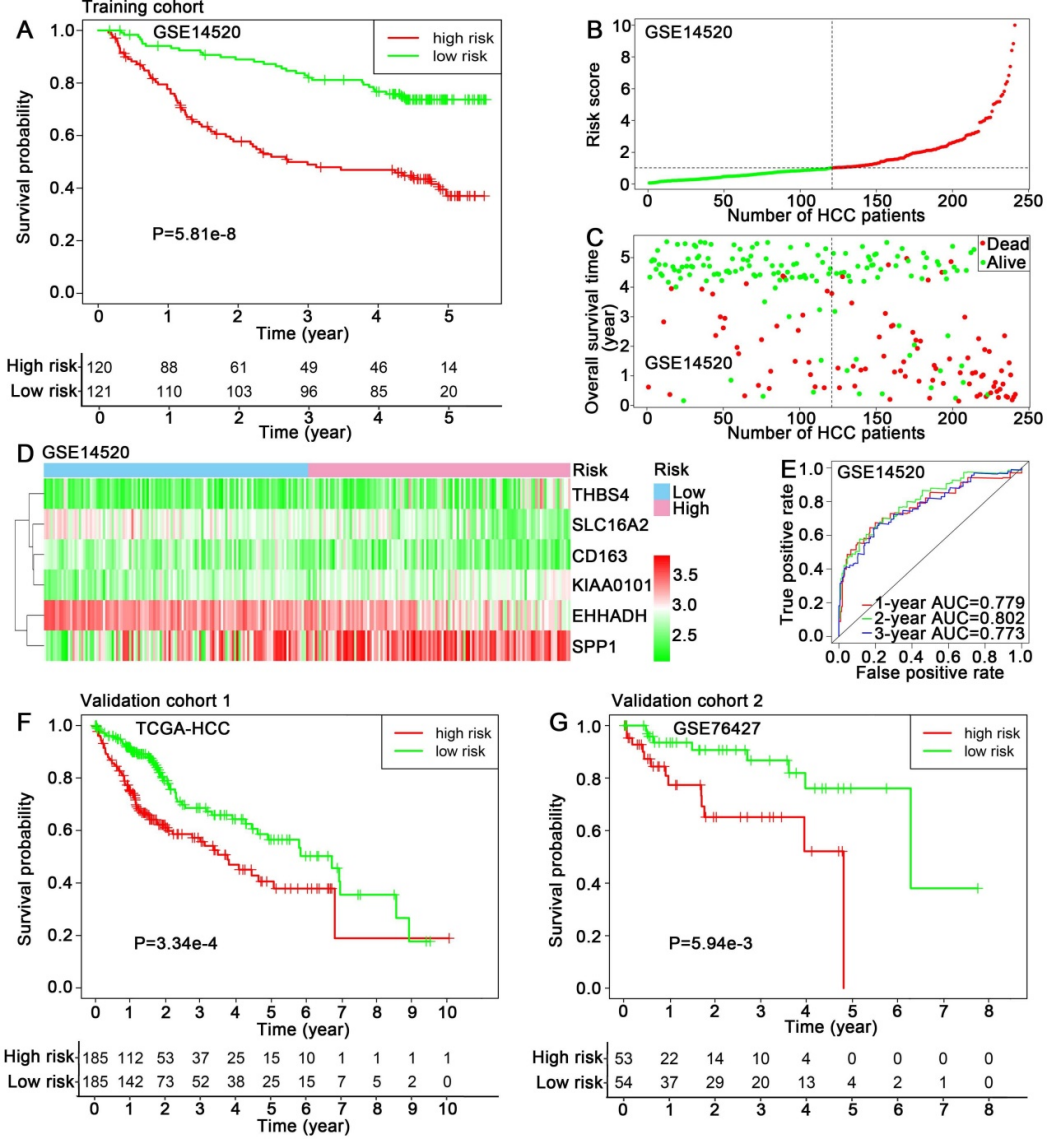
Prognostic assessment of the hub genes-based risk score model. (A) Kaplan-Meier overall survival curve, (B, C) Distribution of risk score, (D) Expression levels of six hub genes and (E) 1, 2, 3-year ROC curves for patients assigned to high- and low risk groups in training cohort GSE14520. Kaplan-Meier overall survival curves for patients assigned to high- and low risk groups in validation cohorts (F) TCGA-HCC dataset and (G) GSE76427.

The training dataset GSE14520 was divided into high-risk group (n=120) and low-risk group (n=121) using the median risk score (1.025) as cut-off value. Kaplan-Meier analysis showed that the patients in high-risk group exhibited a significantly poorer outcome than that in low-risk group (P=5.81e-8) (Figure 4A). The risk scores of HCC patients in GSE14520 were ranked, and the survival status for each HCC patient was also plotted, showing patient mortality in high-risk group was much higher than that in the low-risk group (Figure 4B-C). Time-dependent ROC analyses were performed and 1-, 2-, 3-year AUC were calculated as 0.779, 0.802 and 0.773 (Figure 4E). A heatmap showed the expression profiles of the six hub genes in high- and low-risk HCC patients in GSE14520 (Figure 4D). The prognostic value of the six hub genes were further evaluated in two validation cohorts: TCGA-HCC dataset and GSE76427, showing significant differences in survival outcome between the high-risk and low-risk groups (P=3.34e-4, P=5.94e-3, respectively) (Figure 4F-G). ROC cure and AUC analyses, risk score ranking, survival state plotting and heatmap presentation were also performed and showed in supplemental files (Figure S3A-H).

### Prognostic risk score closely associated with clinicopathological features in HCC

**Figure 5:**
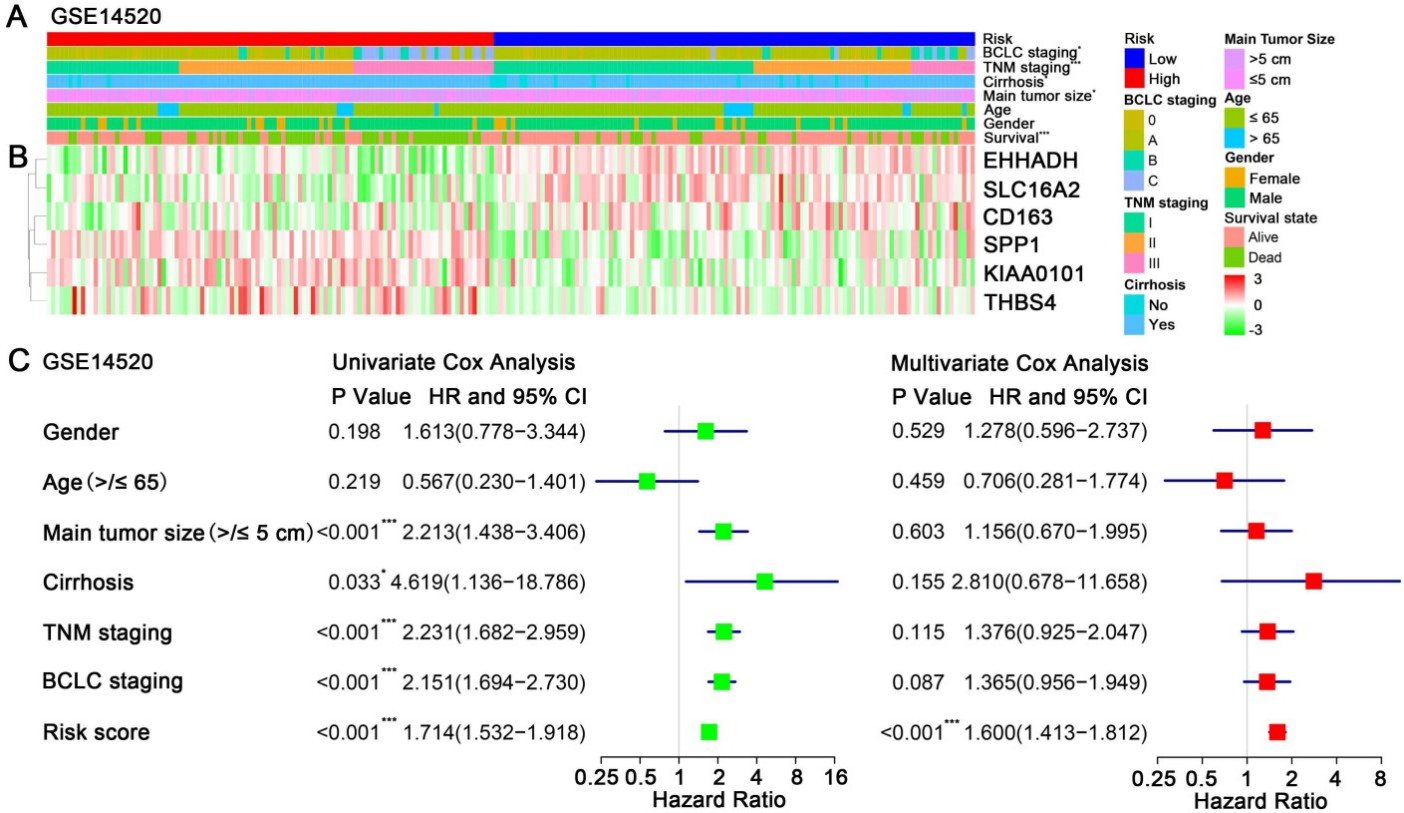
Relationship between the risk score and clinicopathological features. (A) Distribution of risk scores in training dataset GSE14520 stratified by BCLC staging, TNM staging, cirrhosis, main tumor size, age, gender and survival status. (B) Heatmap shows the expression levels of six hub genes in low-risk and high-risk HCC patients in GSE14520. (C) Univariate and multivariate Cox regression analyses of association between risk score, clinicopathological factors and overall survival time of HCC patients in GSE14520.

A heatmap showed the expression value of the six hub genes in high- and low-risk groups in GSE14250 (Figure 5B). Significant differences between the high- and low-risk groups were observed with respect to BCLC staging (P = 0.02), TNM staging (P < 0.001), cirrhosis (P = 0.04), main tumor size (P = 0.04) and survival status (P < 0.001) (Figure 5A). By performing univariate Cox regression analysis, main tumor size, cirrhosis, TNM staging, BCLC staging and risk score were associated with overall survival time. When included these factors into multivariate Cox regression analysis, risk score remained significantly associated with the overall survival time (Figure 5C). Similar results by multivariate Cox regression analysis were also acquired in validation cohorts TCGA-HCC dataset and GSE76427, showing that risk scores were significantly associated the overall survival time (HR = 2.21, P < 0.001; HR = 2.36, P = 0.019; respectively) (Figure S4).

### GSEA for HCC patients in different risk groups

**Figure 6:**
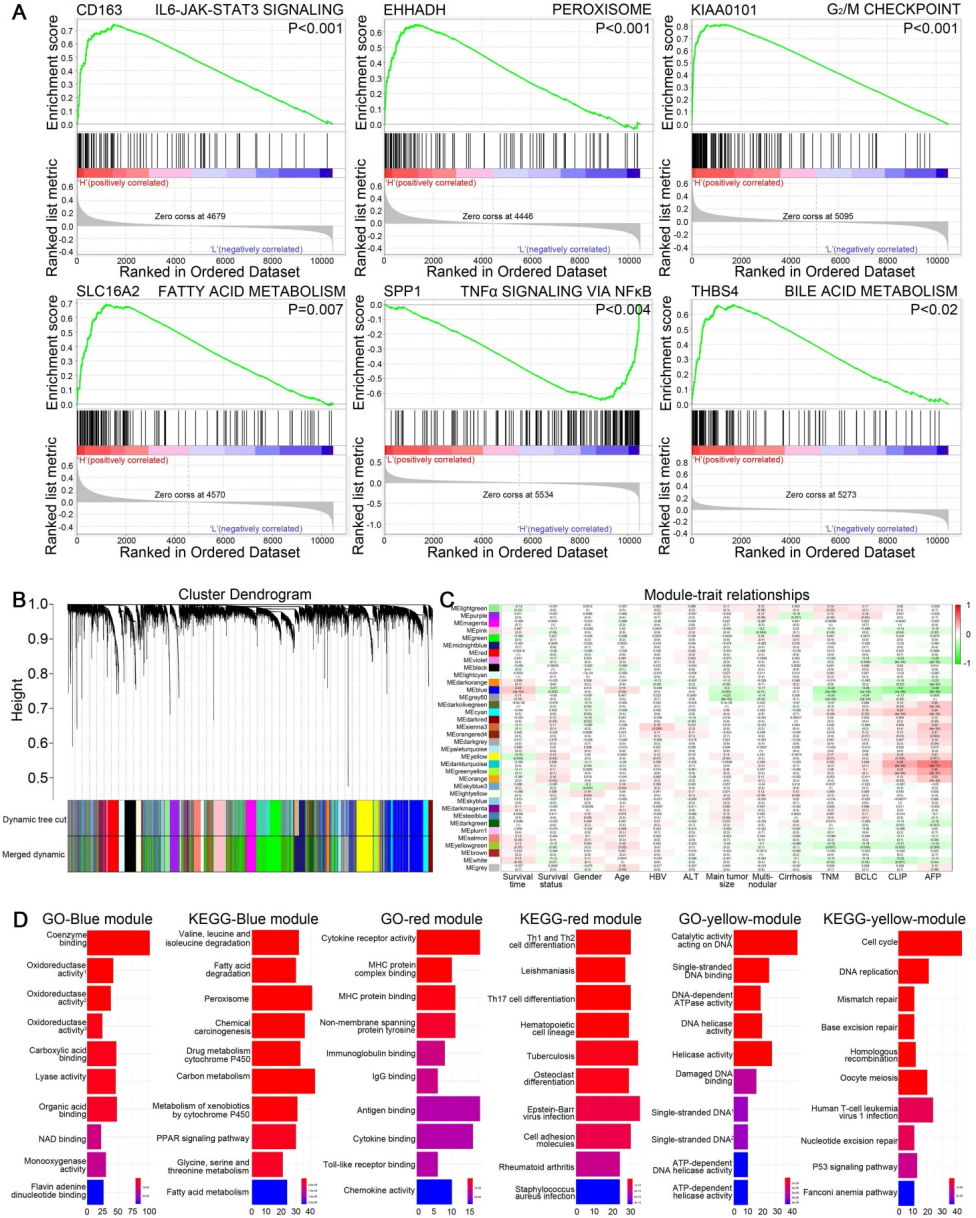
(A) Gene set enrichment analysis of six hub genes in GSE14520. Critical tumorigenesis related signaling pathway ranking among the top three enriched pathways in high expression group of each single hub gene was shown. (B) Dendrogram of genes in GSE14520 (samples with clinical traits) clustered based on a dissimilarity measure (1-TOM). (C) Heatmap of the correlation between module eigengenes and clinical traits in GSE14520. Each cell contains the correlation coefficient and P value. (D) GO and KEGG analyses for genes in blue, red and yellow modules, showing top ten terms according to adjusted p value.

Potential functions of CD163, EHHADH, KIAA0101, SLC16A2, SPP1, THBS4 in HCC were investigated by performing GSEA based on hallmark gene sets, indicating multiple pathways related to tumorigenesis were activated. As shown in Figure 6A, genes in high groups of SLC16A2, EHHADH and THBS4 were respectively enriched in “fatty acid metabolism”, “peroxisome” and “bile acid metabolism” gene sets, which were closely associated with energy metabolism. Meanwhile, “G_2_/M checkpoint” gene set was enriched in high-expression group of KIAA0101, “IL6-JAK-STAT3 signaling” and “TNFα signaling via NFκB” pathways were respectively enriched in the CD163 high-expression and SPP1 low-expression group, all of which were crucial signaling pathways while undergoing oncogenesis and metastasis.

### WGCNA and functional analyses of key modules containing hub genes

WGCNA was performed on GSE14520 dataset to create gene modules associated with clinical traits including survival time, survival state, gender, age, HBV infection, ALT level, main tumor size, multinodular, cirrhosis, TNM staging, BCLC staging, CLIP staging and AFP level. By setting 4 as soft-thresholding power (scale free R^2^=0.85) and 0.25 as cutting height, 36 modules were eventually identified (Figure 6B-C). A heatmap showed the correlation between module eigengenes and clinical traits of HCC (Figure S5A-D). EHHADH, SLC16A2 and SPP1 were contained in blue module. Meanwhile, CD163, KIAA0101 and THBS4 respectively belonged to the red, yellow and grey module. GO and KEGG analyses were conducted on blue, red and yellow gene modules to reveal the potential biological functions. The most significant GO terms and KEGG pathways were shown in Figure 6D, indicating that genes in red module were mainly involved in immune response, genes in yellow module were mainly associated with cell cycle regulation and genes in blue module were mainly related to drug or energy metabolism.

## Discussion

HCC is characterized by high heterogeneity and mortality with the tumorigenesis mechanism remaining to be clarified. The early diagnosis and treatment, as well as follow-up for survival are vital importance for improving the HCC patients' outcome. Since HCC is a complex disease with multiple pathogenic mechanisms caused by various risk factors, it is difficult to predict the outcome with single biomarker. Recently, the high-throughput technology has been rapidly developed and more and more novel potential biomarkers are identified for HCC prognosis 7-10. However, the reported DEGs varied greatly among different researches due to the limited sample size and the inconsistent bioinformatic analyzing methods. Reliable biomarkers that could be applied to most HCC patients are still lacking. Therefore, the sample size of the discovery dataset should be enlarged to eliminate the potential selecting bias and generate more convincing results. Moreover, expression level of the identified hub genes and prognostic value of the established risk model should be further evaluated in other validation cohorts, which could increase the credibility and make the results more reliable.

In the present study, 12 HCC microarray datasets were enrolled as the discovery cohorts, which contained much more samples than previously published works 9-11. Meanwhile, RRA algorithm method was utilized to integrate all the qualified datasets for data analysis, therefore, the robustly DEGs among multiple HCC datasets could be identified for the prognostic model establishment. The HCC patients always show the high mortality rate. Clinical features including tumor size and pathological stage have been used as the indicators to predict the patients' outcome. However, the prognostic results could be varied using these conventional indicators, even in patients with the same tumor size or pathological stage 12. Therefore, we sequentially performed the LASSO regression and multivariate cox regression methods among the selected DEGs and finally established a prognostic model based on six genes (CD163, EHHADH, KIAA0101, SLC16A2, SPP1 and THBS4). Moreover, the multivariate analysis result indicates that the risk score according to the six genes-based model is an independent prognostic factor in HCC, which is also verified in other two validation cohorts, suggesting the potentiality for future clinical application.

Previous studies have reported the individual gene's function during tumorigenesis including vascular invasion 13, 14, sorafenib resistance 15, 16, macrophage activation 17, 18, cell cycle disorder 19-21 and metabolic derangements 22, 23. In our research, the biological functions of the six hub genes were also analyzed by performing GSEA and WGCNA. The results showed that these genes were closely related with energy metabolism, cell cycle regulation, IL6-JAK-STAT3 and TNFα signaling pathways, which play the vital roles while undergoing tumorigenesis.

In conclusion, we screened the genes with the highest differential expressing level among samples from multiple high-throughput HCC datasets, providing the reliable DEGs closely associated with HCC tumorigenesis. Moreover, we constructed a six-gene based prognostic model which could be used to predict the HCC patients' outcome. In addition, the risk score according to the model could be an independent prognostic factor in HCC. Finally, the prognostic value of the six genes-based model has been validated in two independent HCC datasets, indicating the clinical application potentiality. Further research should focus on clarifying how these hub genes contributed to HCC development and validating the prognostic value among larger HCC population.

## Supplementary Material

Supplementary figures and tables.

## Figures and Tables

**Table 1:** Prognostic risk model based on six hub genes in GSE14520

Hub gene	LASSO coefficient	Multivariate Cox regression		
		HR	95% CI	*P* value
CD163	-0.053869857	0.11	0.035-0.38	< 0.001
EHHADH	-0.031966924	0.26	0.089-0.73	0.011
KIAA0101	0.018794088	3.6	0.919-14.14	0.066
SLC16A2	-0.017229864	0.38	0.124-1.17	0.093
SPP1	0.030270674	4.32	2.16-8.63	< 0.001
THBS4	0.11223438	4.78	2.37-9.64	< 0.001

**Table 2:** Real-time PCR primers used for six hub genes and β-actin

Gene	Sequence
CD163	F: 5'-ATTCCTCAGAAAATTCCCATGAGTC-3'
	R: 5'-TCAGAATGGCCTCCTTTTCC-3'
EHHADH	F: 5'-TGCCCTCGGTGATAGAGGAA-3'
	R: 5'-GTCGTACTGATCGCGTTGAC-3'
KIAA0101	F: 5'-GGTGCGGACTAAAGCAGACA-3'
	R: 5'-TTTTTGCCACTTGGGAGTTGG-3'
SLC16A2	F: 5'-GGTAGGAAGGGGCCCTAGAA-3'
	R: 5'-CAGAACCACCCTCTGGTGAC-3'
SPP1	F: 5'-AACGCCGACCAAGGAAAACT-3'
	R: 5'-TGCCCATTTGTTGTTTGGCT-3'
THBS4	F: 5'-CGACCGAGGTTCAACGCA-3'
	R: 5'-ATGTTGGCTCTTCCTGCTCC-3'
β-actin	F: 5'-CTGGAACGGTGAAGGTGACA-3'
	R: 5'-AAGGGACTTCCTGTAACAATGCA-3'

## Abbreviations

HCC: hepatocellular carcinoma
AFP: alpha fetoprotein
GEO: Gene Expression Omnibus
RRA: Robust Rank Aggregation
DEGs: differentially expressed genes
PCR: polymerase chain reaction
GSEA: Gene Set Enrichment Analysis
WGCNA: weighted gene co-expression network analysis
GO: Gene Ontology
KEGG: Kyoto Encyclopedia of Genes and Genomes
ROC: receiver operating characteristic
AUC: area under the ROC curve
TOM: topological overlap matrix
MCT8: monocarboxylate transporter 8
