# Supplementary material for: Identification of hub genes-based predictive model in hepatocellular carcinoma by robust rank aggregation and regression analysis
Source: J Cancer. 2021 Jan 30;12(7):1884–93. doi: 10.7150/jca.52089 (PMC7974519; doi:10.7150/jca.52089)
Supplement: Supplementary file 1 — Supplementary figures and tables. [file jcav12p1884s1.pdf]

Supplemental information

Supplemental figures

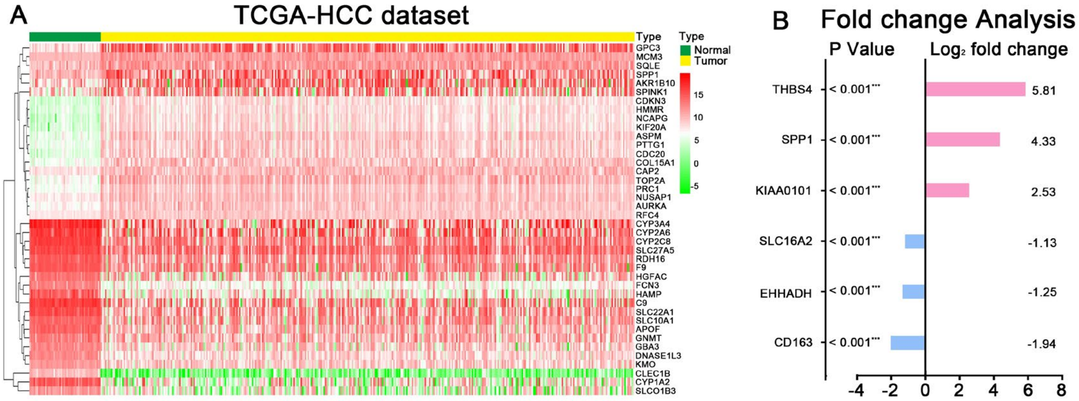

Figure S1. (A) Heatmap of the top 20 up-regulated and top 20 down-regulated DEGs from RRA analyses based on TCGA-HCC dataset. Each row indicated one gene and each column represents one sample. Red represents up-regulation and green represents down-regulation. (B) Logarithmic fold change of the six hub genes between tumor and normal tissues based on TCGA-HCC dataset.

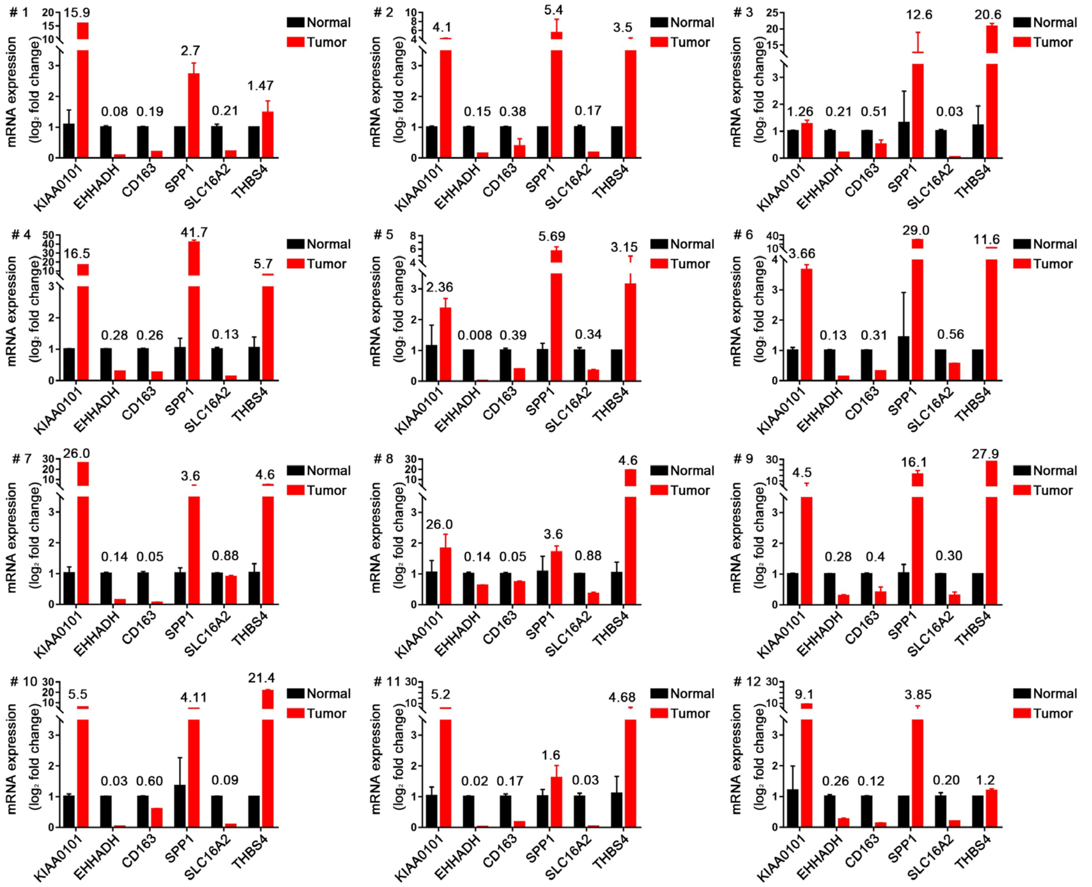

Figure S2. Validation of the six hub genes' expression at transcriptional level in 12 HCC samples and matched normal hepatic samples. The value on each pair of columns shows the log_2fold change for each hub gene between tumor and normal tissues.

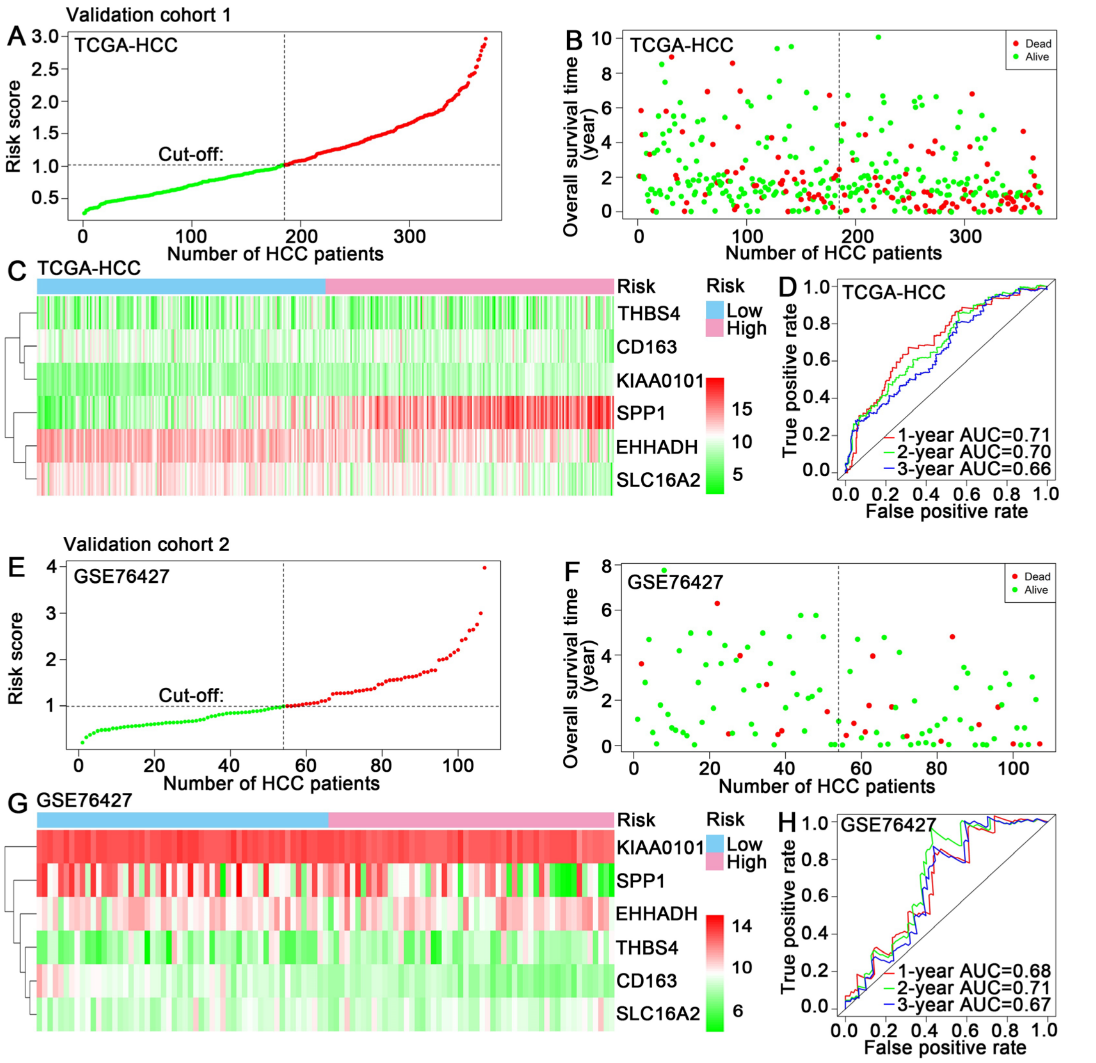

Figure S3 Prognostic assessment of the hub genes-based risk score model. (A, B) Distribution of risk score, (C) Expression levels of six hub genes and (D) 1, 2, 3-year ROC curves for patients assigned to high risk and low risk groups in validation cohort TCGA-HCC dataset. (E, F) Distribution of risk score, (G) Expression levels of six hub genes and (H) 1, 2, 3-year ROC curves for patients assigned to high risk and low risk groups in validation cohort GSE76427.

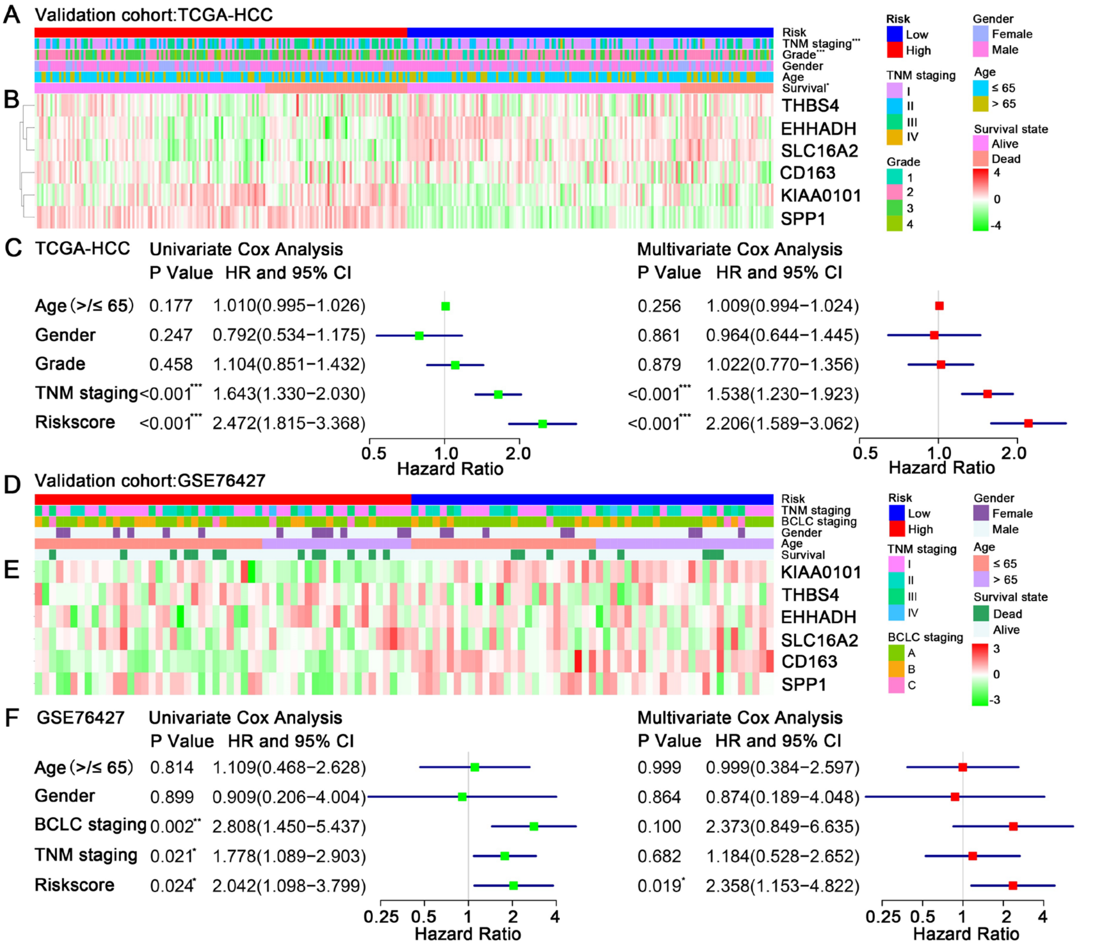

Figure S4. (A) Distribution of risk scores in validation cohort TCGA-HCC dataset stratified by TNM staging, grade, age, gender and survival status. (B) Heatmap shows the expression levels of six hub genes in low-risk and high-risk HCC patients in TCGA-HCC. (C) Univariate and multivariate Cox regression analyses of association between risk score, clinicopathological factors and overall survival time of HCC patients in TCGA-HCC. (D) Distribution of risk scores in validation cohort GSE76427 dataset stratified by TNM staging, BCLC staging, age, gender and survival status. (E) Heatmap shows the expression levels of six hub genes in low-risk and high-risk HCC patients in GSE76427. (F) Univariate and multivariate Cox regression analyses of association between risk score, clinicopathological factors and overall survival time of HCC patients in GSE76427.

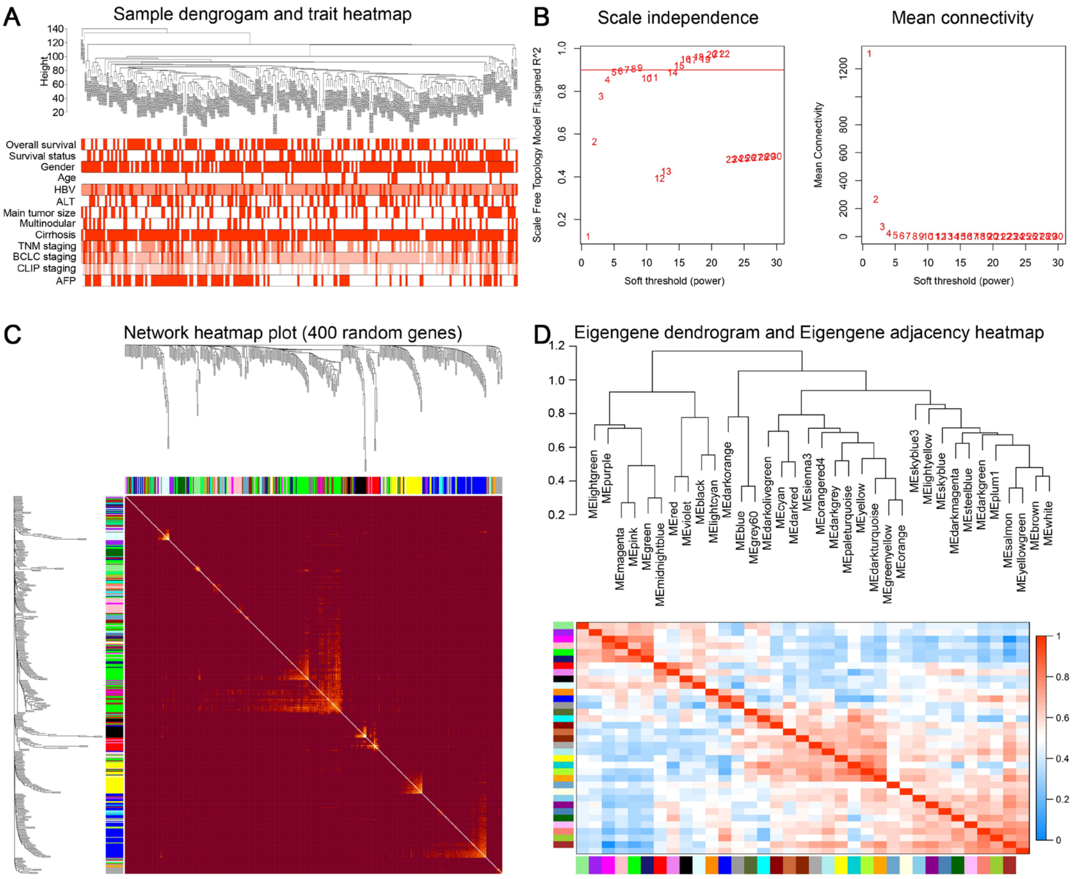

Figure S5. (A) Clustering dendrograms of genes based on GSE14520 (samples with clinical traits) and clinical trait heatmap. (B) Analysis of scale-free fit index (left) and mean connectivity (right) for various soft-thresholding powers. (C) TOM gene co-expression network heatmap plot (based on 400 random genes) (D) Eigengene dendrogram and eigengene adjacency heatmap.

Supplemental table

Table S1 Information of 12 included HCC microarrays

Accession number	Contactor	Platform	Research city	Samples (normal/tumor)	Submission date
GSE25097	Chunsheng Zhang	GPL10687	Boston	249/268	Nov 03, 2010
GSE39791	Sangbae Kim	GPL10558	Houston	72/72	Jul 31, 2012
GSE46408	Yung-Ming Jeng	GPL4133	Taipei	6/6	Apr 26, 2013
GSE57957	Lee Guat Lay Caroline	GPL10558	Singapore	39/39	May 24, 2014
GSE62232	Sandrine Imbeaud	GPL570	Paris	10/81	Oct 09, 2014
GSE64041	Zuzanna Makowska	GPL6244	Basel	65/60	Dec 10, 2014
GSE75271	Toni-Ann Mistretta	GPL570	Houston	5/50	Dec 30, 2015
GSE76427	Surya Pavan Yenamandra	GPL10558	Singapore	52/115	Nov 20, 2016
GSE84005	Jin Song	GPL5175	Beijing	38/38	Jul 05, 2016
GSE84402	Zhuoan Cheng	GPL570	Shanghai	14/14	Jul 14, 2016
GSE14520-1	Xin Wei Wang	GPL571	Bethesda	21/22	Jan 22, 2009
GSE14520-2	Xin Wei Wang	GPL3921	Bethesda	220/225	Jan 22, 2009
